# Lack of transmission among healthcare workers in contact with a case of Middle East respiratory syndrome coronavirus infection in Thailand

**DOI:** 10.1186/s13756-016-0120-9

**Published:** 2016-05-23

**Authors:** Surasak Wiboonchutikul, Weerawat Manosuthi, Sirirat Likanonsakul, Chariya Sangsajja, Paweena Kongsanan, Ravee Nitiyanontakij, Varaporn Thientong, Hatairat Lerdsamran, Pilaipan Puthavathana

**Affiliations:** Bamrasnaradura Infectious Diseases Institute, Ministry of Public Health, Nonthaburi, 11000 Thailand; Faculty of Medical Technology, Mahidol University, Bangkok, Thailand; Faculty of Medicine Siriraj Hospital, Mahidol University, Bangkok, Thailand

**Keywords:** MERS-CoV, Middle East respiratory syndrome, Transmission, Healthcare workers, Healthcare facilities

## Abstract

**Introduction:**

A hospital-associated outbreak of Middle East Respiratory Syndrome Coronavirus (MERS-CoV) was reported. We aimed to assess the effectiveness of infection control measures among healthcare workers (HCWs) who were exposed to a MERS patient and/or his body fluids in our institute.

**Methods:**

A descriptive study was conducted among HCWs who worked with a MERS patient in Bamrasnaradura Infectious Diseases Institute, Thailand, between 18 June and 3 July 2015. Contacts were defined as HCWs who worked in the patient’s room or with the patient’s body fluids. Serum samples from all contacts were collected within 14 days of last contact and one month later. Paired sera were tested for detection of MERS‐CoV antibodies by using an indirect ELISA.

**Results:**

Thirty-eight (88.4 %) of 43 identified contacts consented to enroll. The mean (SD) age was 38.1 (11.1) years, and 79 % were females. The median (IQR) cumulative duration of work of HCWs in the patient’s room was 35 (20–165) minutes. The median (IQR) cumulative duration of work of HCWs with the patient’s blood or body fluids in laboratory was 67.5 (43.7–117.5) minutes. All contacts reported 100 % compliance with hand hygiene, using N95 respirator, performing respirator fit test, wearing gown, gloves, eye protection, and cap during their entire working period. All serum specimens of contacts tested for MERS-CoV antibodies were negative.

**Conclusions:**

We provide evidence of effective infection control practices against MERS-CoV transmission in a healthcare facility. Strict infection control precautions can protect HCWs. The optimal infection control measures for MERS-CoV should be further evaluated.

## Introduction

Middle East respiratory syndrome (MERS) was first reported in 2012 [1]. Clusters of MERS-Coronavirus (MERS-CoV) infection have occurred within extended families, households, and healthcare settings [2]. Human-to-human transmission has been documented including transmission to healthcare workers [3–5]. Although the spread of MERS-CoV is assumed to occur via large droplets and contact, the possibility of airborne transmission has not been excluded [6]. Infection prevention and control guidance for hospitalized patients with MERS from the US Centers for Disease Control and Prevention continues to recommend standard, contact, and airborne precautions. Patients with MERS should be placed in airborne infection isolation room [7]. A recent study demonstrated that MERS-CoV outbreak was associated with exposure to healthcare facilities [8]. The number of healthcare workers (HCWs) acquiring the infection might have resulted from poor infection control measures [5, 9]. Thus, studies to prevent transmission in healthcare setting are critical for the development of control measures. We aimed to assess the effectiveness of infection control measures among HCWs who were exposed to a MERS patient or his body fluids in our institute.

## Methodology

On 18 June 2015, the Bamrasnaradura Infectious Diseases Institute, Ministry of Public Health, Thailand, hospitalized a laboratory-confirmed case of MERS-CoV infection. The patient was a 74-year-old man from Oman who had been admitted to a private hospital 3 days earlier.

The patient had had chest discomfort for one month before admission to the private hospital. He had progressive dyspnea but did not complain of fever. At the private hospital, they found the patient had a temperature of 38.3°c, and chest radiograph revealed an opacity at right lung field. A real-time reverse transcription-polymerase chain reaction (RT-PCR) for MERS-CoV from nasopharyngeal swab showed a positive result on 17 June 2015 (positive for upE and ORF1a assay).

After the MERS-CoV genome was detected, the patient was transferred to our institute. Rapid isolation was done upon receiving the patient. He was placed in an airborne infection isolation room, and precautions to prevent airborne and contact transmission were strictly implemented. Upon the time of admission to our institute, the patient had a frequent cough and oxygen desaturation to 88 %. No fever was detected. He had a respiratory rate of 28 times/min and crepitation without wheezing on lung examination. Bilateral interstitial infiltration was found on his chest radiograph. Repeated RT-PCR for MERS-CoV from nasopharyngeal swab was still positive.

The patient received high flow oxygen and airway care, including sputum suction. Neither aerosol therapy, nor non-invasive ventilation or mechanical ventilator was used. The treatment included intensive care to support vital organ functions, short course intravenous antibiotics, and a 5-day regimen of oseltamivir. He had a stable clinical course characterized by improvement in his oxygenation and resolution of his respiratory symptoms. He was discharged home on 3 July 2015 after PCR for MERS-CoV was negative twice consecutively. Strict infection control measures were performed until the patient was discharged from the hospital.

HCWs who were working with the MERS patient and/or his body fluids were protected by using an N95 respirator that had been fit tested, a gown, disposable gloves, eye protection, and a disposable cap. Hand hygiene was performed before entering and after leaving the patient’s room or after examining his specimens. The patient’s body fluids were examined in the laboratory with a biosafety level 2. Everyone who had been involved with the patient and/or his body fluids had to sign their names and duration of working on a timesheet record.

All materials involved in the case were carefully managed. The waste from the patient and soiled linen were collected in leak-resistant, triple-layered bags. All the exteriors of the bags were disinfected with 70 % alcohol spray. They were destroyed by incineration. The clean linen was collected with triple bagging. They were washed with bleach in a temperature of 70°c for 45 min. Food utensils and dishware were disposable and managed as infectious waste. For the patient’s room, a 0.05 % sodium hypochlorite solution was used to disinfect the room floor daily. The patient’s bed, high-touch surfaces, and room equipment were cleaned by 70 % alcohol twice a day. Contaminated sharps were collected in puncture-resistant containers and incinerated. Clinical specimens were destroyed by incineration.

The investigation focused on HCWs who had worked with the MERS patient or his body fluids at Bamrasnaradura Infectious Diseases Institute, Thailand, from 18 June through 3 July 2015. The body fluids included blood, urine, sputum, nasopharyngeal fluid, and other respiratory secretions. HCWs who had worked in the patient’s room or with the patient’s body fluids were defined as contacts, and identified by the timesheet records. All contacts were assessed retrospectively, but serologic survey was performed prospectively after the patient was discharged. Demographics, type of contact, symptoms within 14 days after contact, and adherence to infection control practices were obtained by questionnaire. Frequency and duration of exposure were retrieved from the timesheet records that were signed by HCWs before and after their works. Serum samples from all contacts were collected within 14 days of last contact and one month later.

Paired sera of HCWs were investigated for the MERS‐CoV antibody by an indirect enzyme-linked immunosorbent assay (EUROIMMUN Medizinische Labordiagnostika AG, Germany) at Faculty of Medicine Siriraj Hospital, Mahidol University, Thailand. The assay protocol of the manufacturer was followed. In brief, a 100 μl volume of the test serum at dilution of 1: 101 was added onto the wells coated with purified S1 antigen of MERS coronavirus in duplicate. Peroxidase labeled rabbit anti-human IgG was used as the secondary antibody and tetramethylbenzidine was used as the substrate. The reaction plate was measured for optical density by a spectrophotometer using the wavelengths of 450 and 630 nm. Positive control, negative control and calibrator human IgG were included in every reaction plate to determine the cut-off value for defining positive, negative or borderline result. Informed consent was obtained from HCWs. The study was reviewed and approved by the Bamrasnaradura Institution Review Board. Results were analyzed by SPSS version 15.0.

## Results

Thirty-eight (88 %) of 43 identified contacts consented to enroll. The mean (SD) age was 38.6 (11.1) years, and 79 % were females. The most frequently exposed groups were laboratory personnel (39 %), nurses (21 %), and radiology technicians (21 %). The most common types of contact were touching the patient, touching the patient’s equipment, and examining clinical specimens. Twenty-one (91 %) of 23 non-laboratory staffs had a distance of contact with the patient of less than 1 m. The median (IQR) frequency and median (IQR) cumulative duration of work of HCWs in the patient’s room were 2 (1–11) times and 35 (20–165) minutes, respectively. The median (IQR) frequency and median (IQR) cumulative duration of work of HCWs with the patient’s blood or body fluids in laboratory were 2 (1–2) times and 67.5 (43.7–117.5) minutes, respectively. All contacts reported 100 % compliance with hand hygiene, using an N95 respirator, performing a respirator fit test, along with wearing a gown, gloves, eye protection, and a cap during their entire working period (Table 1). Three (8 %) HCWs developed symptoms after exposure (rhinorrhea in 1, sore throat in 1, and diarrhea in 1). For the 3 HCWs who developed diarrhea, rhinorrhea and sore throat, the first serum sample was collected at day 5, day 10, and day 12 of their illnesses, respectively. The second sample was collected at day 35, day 40, and day 41 of their illnesses, respectively. All serum specimens of contacts tested for MERS-CoV antibodies were negative whereas those of the patient at day 4, day 9, and day 14 of admission were all positive.

**Table 1:** Characteristics of 38 healthcare workers who were exposed to a MERS patient and/or his body fluids

Characteristics	
Gender, n (%)	
- Male	8 (21.1)
- Female	30 (78.9)
Age, years (Mean ± SD)	38.6 (11.1)
Staff position, n (%)	
- Physician	3 (7.9)
- Nurse	8 (21.1)
- Nursing or patient assistant	3 (7.9)
- Radiology technician	8 (21.1)
- Laboratory personnel	15 (39.4)
- Maid	1 (2.6)
Chronic medical condition, n (%)	
- None	28 (73.7)
- Allergy	5 (13.1)
- Hypertension	2 (5.3)
- Dyslipidemia	2 (5.3)
- Diabetes	1 (2.6)
Type of work, n (%)	
- With patient	23 (60.5)
- With patient’s blood and body fluids	15 (39.5)
Type of contact, n (%)^a^	
- Touching the patient	19 (50.0)
- Touching the patient’s equipment	19 (50.0)
- Examining clinical specimens	15 (39.5)
- Obtaining clinical specimens	5 (13.2)
- Cleaning the patient’s room	2 (5.3)
Activities with the patient, n (%)^a^	
- Positioning	16 (42.1)
- Examining the patient’s blood or body fluids	15 (39.5)
- Feeding	9 (23.7)
- Bed bathing	8 (21.1)
- Radiography	8 (21.1)
- Assessment of vital signs	7 (18.4)
- Physical examination	6 (15.8)
- Medical or intravenous fluid administration	6 (15.8)
- Airway care	4 (10.5)
- Blood, urine or fecal collection	4 (10.5)
- Linen changing	4 (10.5)
- Nasopharyngeal specimen collection	2 (5.3)
Infection control practices while in contact with the patient or his blood and/or body fluids, n (%)	
- Performing hand hygiene	38 (100)
- Wearing an N95 respirator	38 (100)
- Performing a fit test	38 (100)
- Wearing a gown	38 (100)
- Wearing gloves	38 (100)
- Wearing eye protection	38 (100)
- Wearing a cap	38 (100)
Contacts of healthcare workers working in the patient’s room, median (IQR), times	2 (1–11)
Cumulative contact duration of healthcare workers working in the patient’s room, median (IQR), min	35.0 (20.0–165.0)
Distance between healthcare workers and a patient while worked in the patient’s room, n (%)	
- <1 meter	21 (91.3)
- 1–2 meters	2 (8.7)
Contacts of healthcare workers working with the patient’s blood or body fluids in laboratory, median (IQR), times	2 (1–2)
Cumulative contact duration of healthcare workers working with the patient’s blood or body fluids in the laboratory, median (IQR), min	67.5 (43.7–117.5)

^a^More than one response could be given for these characteristics

## Discussions

We describe infection control measures and their effectiveness among HCWs who were exposed to a MERS patient or his body fluids in our institute. Hospital preparedness for receiving MERS patients was designed and exercised before taking the patient based on the administrative control principle. Engineering controls were established in the airborne isolation unit. Personal protective equipment was used according to standard, contact and airborne transmission precaution bases. We did not identify transmission from the patient to any of the contacts who were adherent to the infection control practices. This study supports the US Centers for Disease Control and Prevention recommendations for management of hospitalized patients with MERS-CoV infection including implementing standard, contact and airborne transmission precautions [7]. However, the World Health Organization has recommended the use of surgical masks when caring for MERS patients and particulate respirators for aerosol-generating procedures [10]. Spread of MERS-CoV has been generally assumed to occur via large droplets and contact [6]. There has also been a debate on MERS-CoV respiratory precautions [11]. Although our results support the implementation of airborne transmission precaution, the understanding of the transmission of MERS-CoV is still evolving. The best protective strategy against MERS-CoV should further be assessed.

Some limitations in the study should be acknowledged. First, five contacts denied to participate. Nevertheless, from the infection prevention and control unit’s record, none of them developed any symptoms within 2 weeks after exposure. Second, the use of questionnaires might have caused recall bias. Additionally, adherence to infection control practices was not directly evaluated by observers. However, because all HCWs were negative for MERS-CoV antibodies, the effect of recall on the outcome was probably minimal. Lastly, because of the language and communication problems, we were unable to identify the exact date of illness from the patient or his relatives. Nonetheless, RT-PCR for MERS-CoV was still positive upon the patient’s admission.

We have provided evidence of effective infection control practices against MERS-CoV transmission in a healthcare facility. Strict infection control precautions can protect HCWs. The optimal infection control measures for MERS-CoV should be further evaluated.

## Abbreviations

ELISA: enzyme-linked immunosorbent assay
HCWs: healthcare workers
IQR: interquartile range
MERS: Middle East respiratory syndrome
MERS-CoV: Middle East respiratory syndrome coronavirus
RT-PCR: real-time reverse transcription-polymerase chain reaction
SD: standard deviation
